# Medicina Nautica

**Published:** 1816-01

**Authors:** 


### MEDICINA NAUTICA.

*To the Editors of the Medico-Chirurgical Journal & Review.*

GENTLEMEN,

IF the three following Cases of West Indian Endemic Fever, illustrative of the symptoms and treatment in 70 cases of the same disease, which occurred on board his Majesty's ship SATELLITE, be thought worthy of a place in your excellent Journal, I beg your acceptance of them. They are faithful representations of this dreadful scourge of Europeans, and afford incontestable proofs of the superior efficacy of the depleting system over the stimulating and antiputrescent remedies which were formerly employed to check the ravages of this destructive fever. These proofs have lately been multiplied; but the *corpus historicum* of facts, on so momentous a subject, can hardly be too strong, especially as there is still a literary schism among writers relative to the West Indian fever; one party tracing the concentrated form of the disease to *contagion*, the other party representing it as only a higher *grade* of the marsh or remittent fever of the country.*

Non nostrum inter illos tantas componere lites!

I am, Gentlemen, &c.

P. POWER,
Surgeon R. N.

---

language. No *analysis* of it has yet appeared in the English Journals. We shall endeavour to obtain it, for the purpose of a comprehensive review.

* Vide Bancroft, Chisholm, Burnett, Pym,

CASE 1.—William Arnold, ætatis 26 (seaman), 7th of September, 1814, on passage to the coast of Guiana or Surinam from Barbadoes, is one of six others who presented themselves this morning with violent symptoms of fever. It is to be remarked, that the ship's crew were employed, a few days back, wooding and watering at Prince Rupert's Bay, Dominica. The symptoms are, excessive pain of the head, back, and limbs, with a strong throbbing pulse, blood-shot eyes, countenance flushed, skin preternaturally hot, urine high coloured, great irritation at stomach. — Venæsection to the extent of thirty ounces was immediately had recourse to, and sulph. magnes. ℥j, ol. menth. pip. gutt. ij, to be dissolved in a pint of barley-water, and given in two doses: in the interim, the saline mixture, in a state of effervescence, was given every two hours. — *Vespere.* His stomach has rejected every thing he drank; he has had, however, two watery alvine evacuations: fever high, attended with delirium. Repetatur venæsectio ad uncias xx, and, about an hour after, the cold affusion was liberally used. Ten grains of calomel were administered, and the saline mixture ordered to be continued through the night.

8th. He has had several bilious stools during the night; his fever and delirium greatly diminished; pulse 90 and soft; eyes more lively, with a visible yellowness of the adnata, also of the face and neck. ℞. Pulv. antim. gr. iij, sub. hyd. gr. ij. ft. bolus quartâ q.q. h. s. Continue the mist. salin. ut antea prescrip.—*Vespere.* He perspired freely in the course of the day; but there is a visible exacerbation towards the night, with some return of his delirium; skin hot; head-ache severe; ordered the cold affusion again et habeat medicamenta ut hesterna nocte prescripta.

9th. An evident remission has taken place; has some degree of ptyalism this morning; skin and functions natural. Gave the decoct. cinchonæ in moderate doses, with sago and a little wine as diet.

10th. Medicines and symptoms as yesterday.

11th. A few grains of pulv. rhæi to each dose of the bark.

12th. Omit rhubarb, and continue the bark.

13th. Ut heri.

14th. Convalescent. Discharged to duty 21st of September, 1814.

This is an example of the endemic or bilious continued fever of the country. The abstraction of fifty ounces of blood, a saline cathartic, the cold affusion, and ten grains of calomel, during the first twelve hours, evidently checked the force of the disease, for on the second day there was an *alleviation* of the symptoms. On the third day, the constitution felt the influence of mercury, and there was a complete remission. From this time the convalescence was rapid.

Case 2.—John Humphries, ætatis 28 (seaman), 7th of September, 1814, on passage to Guiana, complains of violent pain in the forehead and eyes, which are much inflamed and intolerant of light. Flushed countenance; skin of a fiery heat; great thirst, with general anxiety and uneasiness; has had three watery alvine evacuations since day-light; distressing pain and sickness of stomach; pulse full, hard, and throbbing. V.S. statim ad uncias triginta, et habeat sol. sul. magn. to be given in divided doses until a full and free evacuation of the bowels is obtained; after which the mist. salin. in actu effervescentiæ secundâ quaque horâ, et decoct. hordei pro potu ordinario.—*Vespere.* In addition to the symptoms in the morning, he is now delirious. Rep. v. s. after which the cold affusion, et hab. bol. sub. hyd. gr. x; mistur. salin. quartis horis. If the heat of the skin should continue, let the body be sponged with vinegar in the course of the night.

8th. He is free from all bad symptoms; his bowels copiously evacuated. Hab. mist. cinch. ter die.

9th. Ut heri.

10th, 11th, and 12th. Convalescent. Discharged 26th September, 1814.

This case of fever was checked by the abstraction of sixty ounces of blood, purgatives, and the cold affusion.

Case 3.—William Hand, ætatis 16 years (boy), 9th September, 1814, complains of violent head-ache. He has a wild and confused appearance; his eyes are wandering, his voice quick and sharp, countenance flushed, skin preternaturally hot, great pain and uneasiness of the stomach, pulse quick and sharp, but not full. Utatur v. s. ad uncias xvj, with the cold affusion. Habeat sub. hyd. gr. x, the stomach being so irritable, that every liquid is rejected.

10th. All his symptoms are aggravated: restraint has now become necessary, which renders him outrageous; he trembles all over, and is frequently shaken with momentary convulsions. Appl. emp. lyttæ capiti raso; and to have the saline mixture in a state of effervescence, which, however, is immediately rejected. An enema was administered, which he retained for some time, and which produced two watery evacuations.—*Vespere.* The heat of the skin and flushed countenance have disappeared, and he is in a comatose and insensible state, with a clammy sweat pervading his whole body. He has had gutt. xij of tinct. opii added to the saline draught, which, however, was immediately rejected. A blister was now applied to the epigastric region: a stimulating glyster was administered, and repeated every two hours, until two stools of a good consistence were procured.

11th. His state of insensibility and low muttering delirium is much worse than yesterday; his sufferings are so great, when he is seized with vomiting after swallowing any liquid, that he struggles hard to avoid taking any. His pulse is low and languid, countenance shrunk and ghastly; skin turning yellow. Some mulled wine was, with difficulty, got down and retained, which induced me to administer xx grains of sub. hyd. in a little sugar, to try and obtain a proper evacuation.—*Vespere.* Three very copious stools, of a good consistence, were obtained: he seems more lively in his countenance, and the irritability is in a great measure subsided; but he is very low and languid, and totally insensible. Wine and spices were now administered in small and frequent doses, and an anodyne was given, as he never closed his eyes since the beginning of his complaint.

12th. He appears approaching fast to dissolution; has the black vomit; eyes fixed, extremities cold; no sign of returning reason: wine and cordials were administered, but he has a total inability to swallow any thing. Clysters of decoct. hordei, Port wine, and tinct. opii, were administered; and blisters applied to the soles of the feet and insides of the thighs.—*Vespere.* An involuntary discharge of urine and watery fæces has taken place; he lies apparently lifeless, and without any hopes on my part of his recovering.

About six o'clock on the morning of the 13th, he knew and spoke to me, and swallowed a little wine, which gave the attendants some hopes of his recovery; however, it proved, as I expected, a deceitful calm, which frequently takes place in this fever, and which I have witnessed in many cases. He expired at seven o'clock A. M. 13th September, 1814.

I learned from some of the people, that this boy was taken ill three days before I discovered that he laboured under any complaint; but the dread of bleeding (having seen the others always on complaining undergo that necessary evacuation) kept him from complaining to me til the disease had got to a dangerous acmé.

In no case did there appear any evidence of the disease being communicated from one individual to another. In some instances, the yellowness of the skin and black vomit answered to what has been observed in the concentrated endemic, or Bulam fever; I am of opinion, however, that the disease varied in degree only, and originated in all cases from the same source, marsh miasmata, and exposure to atmospherical influence.

